# Taking Dogs Into the Office: A Novel Strategy for Promoting Work Engagement, Commitment and Quality of Life

**DOI:** 10.3389/fvets.2019.00138

**Published:** 2019-05-07

**Authors:** Sophie Susannah Hall, Daniel Simon Mills

**Affiliations:** Animal Behaviour, Cognition and Welfare Group, School of Life Sciences, University of Lincoln, Lincoln, United Kingdom

**Keywords:** dogs, office, quality of life, engagement, work performance

## Abstract

Despite growing interest in “take your dog to work” days and the wellbeing benefits associated with interactions with a friendly dog (e.g., animal-assisted activities), there has been little quantification of the benefits of this. We analyzed responses to work-related (work engagement, turnover intention, work-based friendship acuity, social media use, and work-related quality of life) and dog-related (pet dog attachment and dog general health) scales from 749 employees. The predominantly female sample was comprised of 243 employees who brought their dog to work (167 = “often” brought dog to work; 76 = “sometimes” brought dog to work), the remaining 506 did not bring their dog to work. Employees who “often” took their dog to work reported higher than average work engagement on all factors (vigor, dedication, absorption, total), with significant differences reported in comparison to those who “sometimes” (vigor and total) and “never” (vigor, dedication, absorption, total) took their dog to work. Turnover intention was also significantly lower and work-based friendship acuity higher in the group of employees who “often,” compared to “never,” took their dog to work. Benefits of bringing your dog to work were also observed in terms of work-related quality of life, with higher scores on general wellbeing, home-work interface, job career-satisfaction, control at work, working conditions, and overall work quality of life in those who “often” compared to “never” take their dog to work. Employees who “never” took their dog to work reported lower use of social media during break times. We also identified factors which may be important to consider in developing dogs-in-the-workplace policies; dog-demographics including weight (i.e., size), breed-type, and training may be important to consider in defining the ideal office dog and deserve further research. Given the need to improve employee wellbeing and satisfaction to promote effective business performance and economic gain, these results have important implications for office based businesses considering allowing dogs in the workplace.

## Introduction

The evidence in favor of the wellbeing benefits associated with interacting with a pet (typically a dog), has led to a rise in the use of animal-assisted interventions (AAI) in structured visitation programs to organizations (1), such as nursing homes (10, 11), healthcare settings (12–14), and educational institutions (15–17). Historically, dogs in the workplace has typically been limited to allowing trained assistance dogs public access in office-based buildings, to support their owners who may have visual or hearing difficulties (18, 19), or other medical problems such as diabetes. However, with the rise in interest in the value of pet dogs to human health and wellbeing, in both structured interventions and less structured activities (20–24), there is increasing momentum behind “bring your dog to work days” (25, 26).

Being able to bring your pet dog to work potentially offers some intuitively attractive buffers to support psychological wellbeing. The presence of a companion dog has been shown to reduce owner stress, both in the home environment and when completing a challenging task, such as that which may be experienced in a working office (22, 27–29). These benefits are thought to be realized through the calm, non-judgmental constancy that is provided through the presence of a companion animal and through feelings of increased social support (20). As well as offering a direct source of social support (30, 31), dogs are thought to act as social catalysts, increasing communication and friendships with others (21, 32).

If allowing dogs in the workplace improves work-related quality of life and work relations, this may lead to wider benefits, such as increased employee satisfaction, and thus may prove an attractive option for businesses wishing to improve staff retention, employee satisfaction (3), and relations (33, 34), as well as work related quality-of-life (35). Furthermore, with growing concern that increased use of social media can have a negative effect on wellbeing (36, 37), allowing dogs at work might divert employee attention away from social media activities during break periods, further promoting a positive working environment and colleague relations.

However, the number of businesses which allow dogs in the workplace is still very much a minority and there is a lack of strong scientific data to support the benefits of dogs in the office. Current research in this area focuses almost exclusively on qualitative data relating to perceptions of pets in the workplace, or small scale studies (1, 5, 28, 38–41). This research has identified that dogs in the workplace appear to increase perceptions of positive social interactions and the atmosphere at work, but there is a lack of literature which quantifies its direct and indirect effects (42), especially in relation to measures that might impact on productivity. For example, it might be expected that increased positive colleague interactions and improved work atmosphere may improve working vigor, dedication, and commitment to remaining in the place of employment. Additionally, allowing dogs at work may reduce the stress felt by owners at leaving their dog during working hours (43). Dog owning and non-dog owning employees report increased stress over the working day, yet owners who had their dog with them have been known to report less stress over the day (28). Since work-based stress increases cognitive strain and diminishes motivation and memory processes, reducing employee performance (44) and increasing absenteeism and dysfunction (45), this may also have important implications for productivity. Furthermore, some research has also identified that allowing pets in the workplace is associated with a number of challenges and drawbacks, including health and safety, cultural sensitivities, fears, phobias, and disruptions to the working environment (2, 41, 46). As such, the perceived benefits of dogs in the workplace may not be actualized. One key factor which has yet to be scientifically investigated, but which may prove important in determining the extent to which dogs in the office is a useful strategy for promoting work-related outcomes, is to identify whether certain dog-based demographics are associated with different outcomes, for example, it may be that small dogs are less distracting than larger dogs.

With the aim of providing a quantitative assessment of the impact of bringing your dog in to the office on work-related outcomes (employee commitment to work, work placed wellbeing, and social relations) and dog-related outcomes (pet dog attachment and general dog wellbeing), we conducted an internationally advertised survey of dog-owning employees. It was predicted that dog owners who bring their dog in the office would report greater commitment to work, and have enhanced work place wellbeing and social relations, compared to dog owners who do not bring their dog to the office. We also expected that, if allowing employees to take their dog to the office reduced stress associated with leaving the dog at home unattended, employees who took their dog to work would show lower anxious attachment to their dog than those who did not take their dog to the office. With the secondary aim of identifying whether certain dogs make better working companions than others, we assessed whether certain dog-based demographics were related to better work-related outcomes than others.

## Methods

### Survey Design

The survey was designed by the study team and sent for critical appraisal to our study steering group, consisting of four employees and employers working in different office environments. The survey comprised of an introduction and consent page, demographic questions pertaining to the participant, their dog and the nature of their office (items and responses are listed in Table 1) and whether or not they were allowed to take their dog to work (response: yes, no, not sure). Participants were asked to rate their dogs' general health based on the past 3 months on a 5-point Likert scale (1 = poor health, 5 = has had no health issues), this item was used as a rough guide to provide an initial assessment as to whether dogs who went into offices with their owners showed significantly different general health compared to those that did not. Respondents were not informed as to the rationale behind this question. In the following sections, participants were asked questions relating to work-related quality of life, work engagement, turnover intention, social media use, friendship assessment, and pet dog attachment using the following recognized scales and methods of assessment:

**Table 1:** Demographic items and responses across employees.

	**Don't take dog to work (*n* = 506)**	**Take dog to work sometimes (*n* = 76)**	**Take dog to work often (*n* = 167)**
**Employee age**	χ^2^ (10) = 14.38, *p >* 0.05		
18–25 years	18% (91)	18.4% (14)	11.4% (19)
26–35 years	34.4% (174)	46.1% (35)	38.3% (64)
36–45 years	20.0% (101)	14.5% (11)	22.2% (37)
46–55 years	20.0% (101)	11.8% (9)	18.6% (31)
56–65 years	7.5% (38)	7.9% (6)	9.6% (16)
66 years and over	0.2% (1)	1.3% (1)	0% (0)
**Employee gender**	χ^2^ (4) = 1.67, *p* > 0.05		
Female	90.9% (460)	88.2% (67)	89.8% (150)
Male	8.9% (45)	11.8% (9)	9.6% (16)
Non-binary	0.2% (1)	0% (0)	0.6% (1)
**Area of residence**	χ^2^ (8) = 7.59, *p* > 0.05		
UK	92.1% (466)	94.7% (72)	91.0% (152)
North America	6.1% (31)	3.9% (3)	4.8% (8)
Central/South America	0% (0)	0% (0)	0% (0)
Republic of Ireland	0% (0)	0% (0)	0.6% (1)
Europe (excluding British Isles)	1.6% (8)	1.3% (1)	3.6% (6)
Africa	0% (0)	0% (0)	0% (0)
Middle East	0% (0)	0% (0)	0% (0)
South Asia	0% (0)	0% (0)	0% (0)
Asia Pacific	0% (0)	0% (0)	0% (0)
Australia/New Zealand	0.2% (1)	0% (0)	0% (0)
**Organization type**	χ^2^ (10) = 71.56, *p < * 0.001^*^		
Commercial	31.8% (161)	35.5% (27)	41.9% (70)
Not for profit	10.3% (52)	30.3% (23)	27.5% (46)
Government (inc. armed forces)	10.3% (52)	3.9% (3)	3.6% (6)
Healthcare	9.5% (48)	6.6% (5)	4.2% (7)
Education	31.8% (161)	13.2% (10)	15.0% (25)
Other	6.3% (32)	10.5% (8)	7.8% (13)
**Hours in office (typical week)**	χ^2^ (8) = 14.19, *p* > 0.05		
0–1	5.5% (28)	7.9% (6)	10.2% (17)
1–10	10.7% (54)	13.2% (10)	12.0% (20)
11–20	18.6% (94)	10.5% (8)	20.4% (34)
21–30	64.6% (327)	65.8% (50)	57.5% (96)
31 plus	0.6% (3)	2.6% (2)	0% (0)
**Length in current employment**	χ^2^ (12) = 23.21, *p* < 0.03^*^		
<6 months	9.5% (48)	2.6% (2)	3% (5)
6 months−1 year	12.5% (63)	13.2% (10)	9.6% (16)
1–3 years	25.7% (130)	25.0% (19)	25.7% (43)
3–6 years	19.2% (97)	27.6% (21)	16.2% (27)
6–9 years	7.5% (38)	5.3% (4)	13.2% (22)
9–12 years	9.5% (48)	6.6% (5)	11.4% (19)
Over 12 years	16.2% (82)	19.7% (15)	21.0% (35)
**People in office (typical day)**	χ^2^ (10) = 44.62, *p* < 0.001^*^		
Only me	5.5% (28)	2.6% (2)	10.8% (18)
2 people	19.4% (98)	35.5% (27)	31.7% (53)
3–5 people	15.2% (77)	17.1% (13)	15.6% (26)
6–10 people	10.5% (53)	15.8% (12)	8.4% (14)
11–20 people	43.7% (221)	27.6% (21)	24.0% (40)
21 plus people	5.7% (29)	1.3% (1)	9.6% (16)
**Dog age**	χ^2^ (12) = 12.66, *p* > 0.05		
<6 months	3.0% (15)	0% (0)	1.2% (0)
6 months−1 year	8.3% (42)	7.9% (6)	10.2% (17)
1–2 years	20.0% (101)	26.3% (20)	25.7% (43)
3–5 years	30.0% (152)	27.6% (21)	31.7% (53)
6–8 years	19.6% (99)	17.1% (13)	12.6% (21)
9–11 years	12.5% (63)	13.2% (10)	14.4% (24)
12 years plus	6.7% (34)	7.9% (6)	4.2% (7)
**Dog length owned**	χ^2^ (12) = 7.00, *p >* 0.05		
<6 months	6.9% (35)	6.6% (5)	7.8% (13)
6 months−1 year	9.1% (46)	9.2% (7)	14.4% (24)
1–2 years	22.3% (113)	22.4% (17)	23.4% (39)
3–5 years	29.8% (151)	35.5% (27)	27.5% (46)
6–8 years	16.6% (84)	13.2% (10)	13.8% (23)
9–11 years	10.3% (52)	10.5% (8)	9.6% (16)
12 years plus	4.9% (25)	2.6% (2)	3.6% (6)
**Dog sex and neuter status**	χ^2^ (6) = 6.32, *p >* 0.05		
Female–entire	12.8% (65)	18.4% (14)	10.8% (18)
Female–neutered	30.4% (154)	28.9% (22)	33.5% (56)
Male–entire	17.6% (89)	15.8% (12)	22.8% (38)
Male–neutered	39.1% (198)	36.8% (28)	32.9% (55)
**Dog breed type**	χ^2^ (4) = 0.42, *p* > 0.05		
Single (pure) breed	64.8% (328)	64.5% (49)	67.1% (112)
Single cross (e.g., Labrador × Poodle)	20.8% (105)	19.7% (15)	19.2% (32)
Mixed–multiple crosses	14.4% (73)	15.8% (12)	13.8% (23)
**Dog training**	χ^2^ (26) = 45.98, *p* < 0.01^*^		
General obedience (GO)	38.9% (197)	36.8% (28)	33.5% (56)
Kennel club (KC)	2.6% (13)	5.3% (4)	3.0% (5)
Agility (Ag)	0.6% (3)	1.3% (1)	3.0% (5)
Working dog (WD)^a^	1.0% (5)	1.3% (1)	4.2% (7)
Assistance dog (AD)	0% (0)	1.3% (1)	3.0% (5)
Other	1.6% (8)	2.6% (2)	2.4% (4)
None	31.6% (160)	28.9% (22)	26.3% (44)
GO + KC + Ag^b^	2.4% (12)	0% (0)	4.2% (7)
GO + Ag	7.7% (39)	6.6% (5)	9.0% (15)
GO + Ag + WD	1.6% (8)	3.9% (3)	2.4% (4)
GO + WD	3.6% (18)	2.6% (2)	3.6% (6)
GO + KC	7.1% (36)	5.3% (4)	4.8% (8)
AD + Other	0.6% (3)	2.6% (2)	3.6% (6)
KC + Ag	0.8% (4)	1.3% (1)	0% (0)
**Dog weight category**	χ^2^ (6) = 6.24, *p* > 0.05		
<5kg	7.9% (40)	3.9% (3)	9.0% (15)
6–10kg	24.7% (125)	19.7% (15)	20.4% (34)
11–20 kg	29.2% (148)	36.8% (28)	35.9% (60)
21 kg plus	38.1% (193)	39.5% (30)	34.7% (58)

* *Significant chi square test results are discussed in the results section*.

a *Working dog training (e.g., shepherding and gun dog work)*.

b *Categories were recoded to combine training where more employees selected more than one option to ensure each item was mutually exclusive*.

#### Work Related Quality-of-Life (47)

This validated, well used 23-item scale was used to assess quality of life at work (48, 49). The scale is comprised of six factors: general wellbeing (6 items), home-work interface (3 items), job career satisfaction (6 items), control at work (3 items), working conditions (3 items) stress at work (2 items), and total work-related quality of life. Participants answered each question on a 5-point scale (strongly disagree = 1 to strongly agree = 5). Three items are negatively phrased and therefore reverse coded (questions 7, 9, 19). Higher scores indicate greater perceived quality of working life.

#### Utrecht Work Engagement Scale (50, 51)

This validated 9-item scale, comprised of three factors (52, 53), was used to assess employee vigor (3 items), dedication (3 items) and absorption to work (3 items), as well as total work engagement. Participants responded to each item using a 7-point scale (never = 0, always/every day = 6). Mean scores are computed to derive scale scores and total scores, higher scores are indicative of greater work engagement.

#### Turnover Intention Scale (54)

This 6-item scale, with documented reliability and validity (54, 55) evaluates an employee's desire to leave their current job. Participants responded to each item using a 5-point scale (never = 1, always = 5). One item is reverse scored, item scores are added to provide a single total score, higher scores are indicative of greater intention to leave current place of employment.

#### Friendship Assessment Scale (56)

This 6-item scale, with documented validity and reliability (56, 57), was used to assess social support/isolation typically felt at work. Respondents were instructed to consider each item in relation to the past 4 weeks. Items are scored on a 5-point scale (almost always = 0, never = 4), with three items being reverse scored. Higher scores indicate greater friendship acuity. To ensure the scale measured friendships within the workplace, minor amendments were made to the wordings of each item. The amendments are presented in italics: (1) It has been easy to relate to others *in the office*, (2) I felt isolated from other people *in the office*, (3) I had someone to share my feelings with *at work*, (4) I found it easy to get in touch with others *at work*, (5) When with other people *in the office*, I felt separate from them, (6) I felt alone and friendless *at work*. To ensure these minor changes to the item wording did not affect scale reliability Cronbach alphas were computed (α = 0.87) and compared to those reported in the original scale development [α = 0.83; (56)]; scores indicated similar, excellent reliability with the minor word changes.

#### Social Media Use

To assess total amount of time spent using social media (e.g., Facebook, Twitter, Instagram) for non-work activities, we asked participants to approximate the number of minutes spent using these media platforms through three questions; *During break times at work:* (1) Approximately how many minutes do you spend using social media (e.g., Twitter, Facebook, Instagram) for non-work activities on an average day, whilst in the office, but during break times? *During home time:* (2) Approximately how many minutes do you spend using social media (e.g., Twitter, Facebook, Instagram) for non-work activities on an average day, whilst NOT in the office (e.g., at home)? *During work time:* (3) Approximately how many minutes do you spend using social media (e.g., Twitter, Facebook, Instagram) for non-work activities during an average day, during work time (i.e., not during break times)? Participants responded on a 5-point scale (I don't use social media = 0, over 2 h = 4).

#### Pet Attachment Questionnaire (58)

To assess the bond between the owner and their dog we used this validated 26-item scale, comprised of two factors; avoidant and anxious pet attachment. Participants were instructed to answer the questions in relation to their dog. If they owned more than one dog, they were asked to select the dog that they felt the closest to. Items are scored on a 7-point scale (disagree strongly = 1, agree strongly = 7), one item is reverse scored (on the avoidant dimension). Item scores are summed together, higher scores are indicative of greater anxious or avoidant pet attachment.

### Participants and Recruitment

Participants were recruited via press releases, social media and through the University of Lincoln's database of dog owners. The study was carried out in accordance with the recommendations of the British Psychological Society (BPS) Ethical Code of Conduct, with written informed consent from all subjects. All subjects gave written informed consent in accordance with the Declaration of Helsinki. The protocol was approved by the University of Lincoln's College of Science Research Ethics Committee (ID: CoSREC367).

Study advertisements informed that we were interested in understanding the impact of dog ownership on employees and we were looking for dog-owning employees, who currently owned a pet dog and worked in an office environment, to take part in a short survey. For the purpose of the survey, we provided a definition of office as: “a room, set of rooms, or building used for commercial, professional or bureaucratic work. We include teachers working in a school in this definition.” Advertisements specified that we were interested in hearing from employees who did and did not take their dog to work. The opportunity to enter a prize draw for a £50 voucher was offered upon completion of the survey. Interested participants were directed to the survey, hosted via Qualtrics. Recruitment started on 2nd March 2018 and the survey closed on 5th September 2018. A total of 1,055 surveys were started, but 302 did not complete the initial study confirmation statements (I am 18 years or older, I currently own a pet dog, I currently work in an office environment, I visit the office at least once a week, I am aware my responses will be kept confidential and I can contact the research team if I have any questions) (analysis *n* = 749).

### Analysis

Since the aim of the study was to explore the impact of bringing pet dogs in the office, not the impact of allowing dogs in the office, for the first stage of the analysis responses were grouped into those who “never,” “sometimes,” and “often” took their dog to work, rather than those who were allowed to take their dog to work. We first examined participant characteristics between the three “dogs at work” groups (“never,” “sometimes,” “often”) using Chi Square analyses (with Fishers Exact tests where necessary). We examined standardized residuals and adjusted standardized residuals relative to a cut-off point of >2 standard deviations to determine where differences were greatest (7).

Secondly, we assessed whether there was a significant difference in a number of work-related outcomes between employees who took their dog in to the office “often,” “sometimes,” or “never” using univariate or multivariate ANOVAs as appropriate, with Bonferroni corrected *post-hoc* comparisons as necessary. The same approach was used in the third stage of the analysis, which explored significant differences in dog-related outcomes between the three dogs at work groups. In the final stage of the analysis we assessed whether some employees experienced significantly better work-related outcomes than others based upon factors relating to the dog (age, breed-type, gender, length owned, training, and weight) using ANOVAs. Due to the comparably smaller sample size in the number of people who “sometimes” took their dog to work, the categories of “sometimes” and “often” were collapsed to create one group of employees who took their dog to work.

## Results

### Participant Demographics

From the 749 responses, 415 selected that they were not allowed to bring their dog to work (55.4%), 31 of the 274 employees who selected that they were allowed to bring their dog to work indicated they did not bring the dog to work, and 60 selected that they were not sure as they didn't bring their dog to work, these form the “never” group (*n* = 506). Out of the 274 employees who were allowed to bring their dog to work (36.6% of the sample), 243 (88.7% of those allowed) brought their dog to work, with 167 employees “often” (between once a week to every day) bringing their dog, this formed the “often” group, and 76 “sometimes” (between once a month to a few times a month) bringing their dog to work, this formed the “sometimes” group.

Females, residing in the UK, were the greatest responders to the survey (Table 1). The most frequently represented age category was 26–35 year olds, who worked for commercial organizations. The majority of employees worked 21–30 h in the office on a typical week and had been in their current place of employment for between 1 and 3 years.

The significance of associations between taking your dog to work and demographic factors are reported in Table 1, where we report the standardized and adjusted residuals for the tests which were statistically significant.

#### Organization Type

Significantly more people than expected take their dog to work if they work for a not-for-profit organization and less people than expected take their dog to work if they are employed in the education sector. Indeed, the number of employees who “never” take their dog to work were fewer than expected in not-for-profit organizations (standardized residual: −3.3; adjusted residual: −6.3) and higher than expected in education (standardized residual: 2.5; adjusted residual: 5.1). Employees who “sometimes” take their dog were greater than expected in not-for-profit organizations (standardized residual: 3.1; adjusted residual: 3.5) and lower than expected in education (standardized residual: −2.2; adjusted residual: −2.7). Additionally, the number of employees who “often” take their dog were greater than expected in those working for not-for-profit organizations (standardized residual: 3.7; adjusted residual: 4.5) and lower in education (standardized residual: −2.8; adjusted residual: −3.7).

#### Length in Current Employment

Working in the current place of employment for <6-months was associated with significantly lower than expected counts of “often” taking your dog to work (standardized residual: −2.1; adjusted residual: −2.4), whereas working in the current place of employment for 6–9 years was associated with higher than expected counts of “often” taking your dog to work (standardized residual: 2.0; adjusted residual: 2.4).

#### Number of People in the Office

Bringing your dog to work regularly was significantly associated with working in smaller offices (fewer employees). The number of employees who “never” bring their dog to work were lower than expected if 2 people are in the office (standardized residual: −2.0; adjusted residual: −4.1), and higher than expected if 11–20 people work in the office (standardized residual: 2.2; adjusted residual: 4.9). Employees who “often” bring their dog to work were greater than expected if 1 (standardized residual: 2.2; adjusted residual: 2.6), and 2 people (standardized residual: 2.1; adjusted residual: 2.7) worked in the office and lower than expected if 11–20 people (standardized residual: −2.9; adjusted residual: −4.1) worked in the office.

#### Dog Training

Bringing your dog to work regularly may be associated with having a dog that has received assistance dog training and working dog training (e.g., shepherding or gun dog work). Employees who “never” took their dog to work had significantly lower than expected counts for their dog receiving assistance dog training (standardized residual: −2.0; adjusted residual: −3.5). However, there were no clear associations for “sometimes” and “often.” Employees who “often” took their dog to work had higher than expected counts for their dog receiving working dog training (standardized residual: 2.4; adjusted residual: 2.8), although there were no clear associations for “sometimes” and “never.”

### Work-Related Outcomes

In the second stage of the analysis we assessed differences in work-related outcomes between the three dogs at work groups to evaluate the impact of bringing your dog to work on perceived work engagement, turnover intention, work-based friendships, use of social media, and work-related quality of life. Descriptive statistics and, where possible, comparison to norm scores are presented in Table 2. It should be pointed out, that whilst norm scores are computed based on a sample in which males and females were similarly represented, our sample is predominately female.

**Table 2:** Descriptive statistics for work engagement, work-related quality of life (QoL), turnover intention, friendship acuity and social media use, and pet dog attachment, across the three dogs at work groups, including comparison to norm scores.

	**Never (*n* = 506)**	**Sometimes (*n* = 76)**	**Often (*n* = 167)**	**Norm data**
**Work engagement**	Mean ± SEM			
Vigor	3.05 ± 0.06	3.51 ± 0.15	3.87 ± 0.10	4.01 ± 0.01^*^
Dedication	3.75 ± 0.07	4.09 ± 0.15	4.52 ± 0.09	3.88 ± 0.01
Absorption	3.75 ± 0.06	4.09 ± 0.14	4.47 ± 0.08	3.35 ± 0.01
Total	3.52 ± 0.06	3.91 ± 0.13	4.28 ± 0.08	3.74 ± 0.01
**Work related QoL**				
General well-being	3.47 ± 0.03	3.69 ± 0.08	3.79 ± 0.05	3.62 ± 0.02^**^
Home-work interface	3.39 ± 0.04	3.70 ± 0.10	4.00 ± 0.06	3.48 ± 0.03
Job-career satisfaction	3.61 ± 0.03	3.79 ± 0.08	3.96 ± 0.05	3.50 ± 0.03
Control at work	3.34 ± 0.04	3.66 ± 0.09	4.00 ± 0.05	3.43 ± 0.03
Working conditions	3.82 ± 0.03	3.95 ± 0.08	4.21 ± 0.04	3.45 ± 0.03
Stress at work	2.55 ± 0.04	2.75 ± 0.13	2.75 ± 0.07	2.69 ± 0.03
Overall work QoL	3.52 ± 0.04	3.78 ± 0.10	4.02 ± 0.07	3.44 ± 0.02
**Turnover intention**	17.82 ± 0.19	16.96 ± 0.53	15.19 ± 0.35	max score 75
**Friendship acuity**	17.08 ± 0.24	19.35 ± 0.47	19.19 ± 0.41	<15 = low, >19 = high
**Social media use**				
During work	1.00 ± 0.04	1.26 ± 0.12	1.17 ± 0.08	n/a
During breaks	1.17 ± 0.03	1.38 ± 0.11	1.35 ± 0.06	n/a
At home	2.54 ± 0.04	2.63 ± 0.12	2.68 ± 0.08	n/a
**Pet dog attachment**				
Anxious	35.40 ± 0.51	36.26 ± 1.33	32.37 ± 0.89	n/a
Avoidant	17.95 ± 0.24	17.49 ± 0.61	17.14 ± 0.41	n/a
**Dog health score**	4.23 ± 0.40	4.24 ± 0.10	4.35 ± 0.07	5 = No health issues

* (59), n = 9,679;

** *(60), n = 3,797*.

#### Work Engagement

There was a significant difference in work engagement across the three “dogs at work” groups, in terms of:

Vigor: *F*_(2, 746)_ = 22.81, *p* < 0.001, η_p_^2^ = 0.058, being significantly higher in individuals who “often” (*p* < 0.001) and “sometimes” (*p* = 0.02) took their dog to work, compared to those who “never” took their dog to work.
Dedication: *F*_(2, 746)_ = 19.18, *p* < 0.001, η_p_^2^ = 0.049, being significantly higher in those who “often” took their dog to work compared to “never” (*p* < 0.001).
Absorption: *F*_(2, 746)_ = 19.03, *p* < 0.001, η_p_^2^ = 0.049, being significantly higher in those who “often” took their dog to work compared to “never” (*p* < 0.001).
Total work engagement: *F*_(2, 746)_ = 24.46, *p* < 0.001, η_p_^2^ = 0.062, which was significantly higher in those who often (*p* < 0.001) and “sometimes” (*p* < 0.04) took their dog to work, compared to “never.”

Comparison of our data, based on a mainly female sample, to normed data, based on male and female samples, showed that for employees who “never” and “sometimes” took their dog to work, vigor, dedication, absorption, and total work engagement scores fell in the “average” category (Table 2). For employees who “often” took their dog to work, levels of absorption fell in the “high” category, levels of vigor, dedication and total work engagement were classed as “average” (59).

#### Turnover Intention

There was a significant difference in turnover intention between the three “dogs at work” groups *F*_(2, 746)_ = 21.61, *p* < 0.001, η_p_^2^ = 0.055. Those who “often” took their dog to work had significantly lower intentions to leave than those who “sometimes” (*p* < 0.02) and “never” took their dog to work (*p* < 0.01). Although it was not possible to compare scores to normed data for this scale, given a maximum possible score of 75, the average for each group seemed to indicate that turnover intentions could be considered low in all groups (see Table 2).

#### Friendship Acuity

There was a significant difference in friendship acuity between the three “dogs at work” groups *F*_(2, 746)_ = 14.05, *p* < 0.001, η_p_^2^ = 0.036. Those who “often” took their dog to work reported significantly higher friendship acuity than those who “never” took their dog to work (*p* < 0.01). Normed data have not been reported for this scale, however, scoring guidelines suggest that employees who “sometimes” and “often” bring their dogs to work have high work-based friendship acuity, whereas employees who “never” take their dog to work have more typical friendship acuity (Table 2).

#### Social Media Use

There was no significant difference in frequency of social media use during work hours *F*_(2, 746)_ = 3.04, *p* = 0.05, η_p_^2^ = 0.008, or when at home *F*_(2, 746)_ = 1.11, *p* = 0.33, η_p_^2^ = 0.003, between the three “dogs at work” groups. However, there was a significant difference in social media use during break time at work *F*_(2, 746)_ = 4.68, *p* = 0.01, η_p_^2^ = 0.012. In contrast to what may be expected, *post-hoc* comparisons revealed that those who “never” took their dog to work used social media less in break times than those who “often” took their dog to work (*p* = 0.03, Table 2).

#### Work-Related Quality of Life

There was a significant difference in work-related quality of life across the three “dogs at work” groups. This was evidenced across the work-related quality of life factors:

General wellbeing: *F*_(2, 746)_ = 13.25, *p* < 0.001, η_p_^2^ = 0.034, with *post-hoc* comparisons revealing significantly higher scores in those who “often” compared to “never” took their dog to work (*p* < 0.001). Mean scores in our predominately female sample, were similar to normed data, based on male and female samples, across the groups (Table 2).
Home-work interface: *F*_(2, 746)_ = 30.11, *p* < 0.001, η_p_^2^ = 0.075, with significantly higher scores in those who “often” compared to “sometimes” (*p* < 0.05) and “never” took their dog to work (*p* < 0.02) and higher scores in those who “sometimes” compared to “never” took their dog to work (*p* < 0.02). Comparison of means revealed scores on home-work interface in employees who took their dog to work were higher than the norm (based on male and female samples), with employees who “often” took their dog to work falling in the upper 75th percentile.
Job and career satisfaction: *F*_(2, 746)_ = 16.47, *p* < 0.001, η_p_^2^ = 0.042, with significantly higher scores in those who “often” compared to “never” took their dog to work (*p* < 0.001). Mean scores were higher than the male and female normed data for our predominately female employees who “sometimes” and “often” took their dog to work.
Control at work: *F*_(2, 746)_ = 41.47, *p* < 0.001, η_p_^2^ = 0.100, with significantly higher scores in those who “often” compared to “sometimes” (*p* < 0.01) and “never” took their dog to work (*p* < 0.01) and higher scores in those who “sometimes” compared to “never” took their dog to work (*p* < 0.01). Mean scores were higher than the male and female normed data for our mainly female employees who “sometimes” and “often” took their dog to work.
Working conditions: *F*_(2, 746)_ = 22.53, *p* < 0.001, η_p_^2^ = 0.057, with significantly higher scores in those who “often” compared to “sometimes” (*p* < 0.01) and “never” took their dog to work (*p* < 0.01). There was no statistically significant difference between “sometimes” and “never,” although mean scores were higher than the norm for employees who “sometimes” and “often” took their dog to work; employees who “often” took their dog to work falling in the upper 75th percentile.
Stress at work: *F*_(2, 746)_ = 3.30, *p* < 0.04, η_p_^2^ = 0.009, however, *post-hoc* comparisons failed to identify statistically significant differences between the three groups and mean scores were similar to normed data across the groups. As such this result is not considered a significant finding.
Overall work quality of life: *F*_(2, 746)_ = 19.98, *p* < 0.001, η_p_^2^ = 0.051, being significantly higher in those who “often,” compared to “never,” took their dog to work (*p* < 0.01). Mean scores were higher than the norm (based on male and female samples) for the employees in this study who “sometimes” and “often” took their dog to work, with employees who “often” took their dog to work falling in the upper 80th percentile.

### Dog-Related Outcomes

#### Pet Dog Attachment

There was a significant difference in anxious attachment to the pet dog between the three “dogs at work” groups, *F*_(2, 746)_ = 4.93, *p* < 0.01, η_p_^2^ = 0.013. As predicted, those who “often” took their dog to work showed significantly lower anxious attachment than those who “sometimes” (*p* < 0.05) and “never” took their dog to work (*p* < 0.02, Table 2). There was no statistically significant difference in avoidant attachment, *F*_(2, 746)_ = 1.53, *p* > 0.05, between the groups.

#### Dog Health Issues

There was no significant difference in self-reported dog health scores between employees who “never,” “sometimes,” and “often” took their dog to work *F*_(2, 746)_ = 1.20, *p* > 0.05. Mean scores across the three groups were close to 5 (no health issues) (Table 2).

### The Impact of Dog Demographics on Work-Related Outcomes

#### Work Engagement

Dog weight significantly affected work engagement for employees who took their dog to work. Both “absorption” *F*_(3, 242)_ = 3.29, *p* = 0.02, η_p_^2^ = 0.04, and “total” work engagement *F*_(3, 242)_ = 3.42, *p* = 0.02, η_p_^2^ = 0.04, were significantly higher in employees who owned a ≤5 kg (n = 18) dog compared to a ≥21 kg dog (n = 88) (*ps* < 0.05) (see Table 3). Dog weight also significantly affected “vigour” *F*_(3, 242)_ = 2.76, *p* = 0.04, η_p_^2^ = 0.03, *post-hoc* comparisons revealed a similar trend to that with “absorption” and “total” work engagement, but Bonferroni comparisons did not identify specific significant differences.

**Table 3:** Descriptive statistics for work engagement in employees who take their dog to their office as a function of dog-based demographics.

	**Vigor**	**Dedication**	**Absorption**	**Total**
**Dog factors**	(Mean ± SEM)				
**Age**				
<6 months	2.85 ± 0.93	4.15 ± 0.91	3.65 ± 0.78	3.55 ± 0.80
6 months−1 year	3.26 ± 0.27	4.01 ± 0.26	4.24 ± 0.23	3.83 ±0.23
1–2 years	3.97 ± 0.16	4.41 ± 0.16	4.54 ± 0.14	4.31 ± 0.14
3–5 years	3.76 ± 0.15	4.33 ± 0.15	4.16 ± 0.12	4.09 ± 0.13
6–8 years	3.72 ± 0.22	4.55 ± 0.22	4.30 ± 0.19	4.19 ± 0.19
9–11 years	3.65 ± 0.23	4.49 ± 0.22	4.43 ± 0.19	4.20 ± 0.19
12 years plus	3.95 ± 0.36	4.58 ± 0.35	4.65 ± 0.31	4.39 ± 0.31
**Breed type**				
Single breed	3.69 ±0.10	4.35 ± 0.10	4.32 ± 0.08	4.12 ± 0.08
Single cross	3.83 ± 0.19	4.46 ± 0.18	4.43 ± 0.16	4.24 ± 0.16
Mixed	3.94 ± 0.22	4.44 ± 0.21	4.38 ± 0.18	4.25 ± 0.19
**Sex and neuter status**				
Female entire	3.98 ± 0.23	4.64 ± 0.22	4.67 ± 0.19	4.43 ± 0.19
Female neutered	3.76 ± 0.15	4.32 ± 0.14	4.30 ± 0.12	4.14 ± 0.12
Male entire	3.56 ± 0.18	4.11 ± 0.18	4.17 ± 0.15	3.94 ± 0.15
Male neutered	3.77 ± 0.14	4.51 ± 0.14	4.37 ± 0.12	4.23 ± 0.12
**Length owned**				
<6 months	3.68 ± 0.31	4.44 ± 0.30	4.35 ± 0.26	4.35 ± 026
6 months−1 year	3.38 ± 0.23	4.07 ± 0.23	4.29 ± 0.19	4.29 ± 0.19
1–2 years	3.98 ± 0.17	4.37 ± 0.17	4.53 ± 0.14	4.53 ± 0.14
3–5 years	3.66 ± 0.15	4.30 ± 0.15	4.10 ± 0.12	4.10 ± 0.12
6–8 years	3.85 ± 0.22	4.64 ± 0.22	4.40 ± 0.19	4.41 ± 0.19
9–11 years	3.54 ± 0.26	4.45 ± 0.26	4.42 ± 0.22	4.42 ± 0.22
12 years plus	4.80 ± 0.46	5.12 ± 0.45	5.07 ± 0.39	5.07 ± 0.39
**Training**				
General obedience (GO)	3.66 ± 0.14	4.43 ± 0.13	4.33 ± 0.12	4.15 ± 0.12
Kennel club (KC)	3.88 ± 0.44	4.01 ± 0.42	4.38 ± 0. 37	4.08 ± 0.37
Assistance dog (AD)	3.33 ± 0.53	4.10 ± 0.52	3.73 ± 0.46	3.73 ± 0.46
Agility (Ag)	3.70 ± 1.31	4.70 ± 1.27	3.70 ± 1.12	4.00 ± 1.12
Working dog (WD)	4.32 ± 0.46	4.79 ± 0.52	4.63 ± 0.40	4.58 ± 0.40
Other	4.10 ± 0.53	4.95 ± 0.15	4.88 ± 0.46	4.65 ± 0.46
None	3.71 ± 0.16	4.27 ± 0.48	4.31 ± 0.14	4.10 ± 0.14
GO + KC + Ag	4.55 ± 0.49	5.20 ± 4.81	4.77 ± 0.42	4.84 ± 0.43
GO + Ag	4.27 ± 0.29	4.87 ± 0.28	4.74 ± 0.25	4.62 ± 0.25
GO + Ag + WD	3.38 ± 0.49	3.74 ± 0.48	4.04 ± 0.42	3.71 ± 0.43
GO + WD	4.20 ± 0.46	4.83 ± 0.45	4.36 ± 0.40	4.48 ± 0.40
GO + KC	3.10 ± 0.38	3.76 ± 0.36	4.00 ± 0.32	3.63 ± 0.32
AD + Other	3.41 ± 0.46	3.96 ± 0.45	4.25 ± 0.40	3.89 ± 0.40
KC + Ag	2.70 ± 1.31	4.00 ± 1.27	4.30 ± 1.12	3.70 ± 1.12
**Weight**				
<5kg	4.35 ±.031	4.94 ± 0.30	4.94 ± 0.26	4.76 ± 0.26
6–10 kg	3.56 ± 0.19	4.30 ± 0.18	4.17 ± 0.16	4.02 ± 0.16
11–20 kg	3.93 ± 0.14	4.53 ± 0.14	4.49 ± 0.12	4.32 ± 0.12
21 kg plus	3.57 ± 0.14	4.18 ± 0.14	4.19 ± 0.12	3.98 ± 0.12

#### Turnover Intention

One dog-based factor, dog training, significantly affected turnover intention for employees who took their dog to work, *F*_(13, 242)_ = 2.01, *p* = 0.02, η_p_^2^ = 0.10. Turnover intention was higher if the dog had received general obedience training combined with agility and working dog training (*n* = 7) compared to employees whose dog had received general obedience training combined with agility and Kennel Club training (*n* = 7) (*p* < 0.01) (see Table 4),although it should be noted the sample size in these sub-groups is small.

**Table 4:** Descriptive statistics for social media use, friendship acuity and turnover intention, in employees who take their dog to their office as a function of dog-based demographics.

	**Social media used in breaks**	**Friendship acuity**	**Turnover intention**
**Dog factors**	(Mean ± SEM)		
**Age**			
<6 months	1.50 ± 0.61	16.50 ± 3.48	16.00 ± 3.30
6 months−1 year	1.43 ± 0.18	18.57 ± 1.03	16.09 ± 0.97
1–2 years	1.41 ± 0.11	19.98 ± 0.62	15.60 ± 0.59
3–5 years	1.57 ± 0.10	18.62 ± 0.57	15.73 ± 0.54
6–8 years	1.12 ± 0.15	20.18 ± 0.84	15.32 ± 0.80
9–11 years	1.18 ± 0.15	19.44 ± 0.84	15.50 ± 0.80
12 years plus	1.00 ± 0.24	17.85 ± 1.37	17.62 ± 1.30
**Breed type**			
Single breed	1.46 ± 0.07	19.34 ± 0.39	15.93 ± 0.37
Single cross	1.30 ± 0.13	18.53 ± 0.72	15.19 ± 0.68
Mixed	1.03 ± 0.15	19.77 ± 0.83	15.66 ± 0.79
**Sex and neuter status**			
Female entire	1.56 ± 0.15	19.31 ± 0.88	16.41 ± 0.82
Female neutered	1.29 ± 0.10	19.45 ± 0.56	15.19 ± 0.52
Male entire	1.54 ± 0.12	19.18 ± 0.70	15.16 ± 0.65
Male neutered	1.25 ± 0.10	19.06 ± 0.54	16.36 ± 0.51
**Length owned**			
<6 months	0.94 ± 0.20	19.72 ± 1.16	15.78 ± 1.10
6 months−1 year	1.35 ± 0.15	19.19 ± 0.88	15.74 ± 0.84
1–2 years	1.52 ± 0.11	20.09 ± 0.66	15.95 ± 0.63
3–5 years	1.56 ± 0.10	18.26 ± 0.58	15.86 ± 0.55
6–8 years	1.09 ± 0.15	19.33 ± 0.86	15.73 ± 0.82
9–11 years	1.29 ± 0.18	19.17 ± 1.00	15.46 ± 0.96
12 years plus	0.88 ± 0.30	21.15 ± 1.74	14.13 ± 1.66
**Training**			
General obedience (GO)	1.39 ± 0.10	19.52 ± 0.54	15.56 ± 0.49
Kennel club (KC)	1.33 ± 0.29	18.56 ± 1.64	14.89 ± 1.51
Assistance dog (AD)	0.67 ± 0.36	16.00 ± 2.01	19.33 ± 1.85
Agility (Ag)	1.00 ± 0.88	22.00 ± 4.93	9.00 ± 4.52
Working dog (WD)	1.00 ± 0.31	19.75 ± 1.74	15.13 ± 1.60
Other	1.00 ± 0.36	21.50 ± 2.01	14.67 ± 1.85
None	1.53 ± 0.11	19.20 ± 0.61	16.23 ± 0.56
GO + KC + Ag	1.57 ± 0.33	19.57 ± 1.86	12.29 ± 1.71
GO + Ag	1.10 ± 0.20	19.30 ± 1.10	14.65 ± 1.01
GO + Ag + WD	1.43 ± 0.33	19.57 ± 1.86	21.43 ± 1.71
GO + WD	1.75 ± 0.31	20.63 ± 1.74	14.50 ± 1.60
GO + KC	1.25 ± 0.25	19.42 ± 1.42	15.67 ± 1.31
AD + Other	1.25 ± 0.31	14.63 ± 1.74	15.75 ± 1.60
KC + Ag	1.00 ± 0.88	22.00 ± 4.93	21.00 ± 4.52
**Weight**			
<5kg	1.61 ± 0.21	19.50 ± 1.15	14.83 ± 1.10
6–10 kg	1.45 ± 0.13	19.86 ± 0.70	15.98 ± 0.67
11–20 kg	1.36 ± 0.09	19.84 ± 0.52	15.84 ± 0.50
21 kg plus	1.27 ± 0.09	18.25 ± 0.52	15.70 ± 0.50

#### Friendship Acuity

Dog-based factors did not significantly affect friendship acuity (see Table 4).

#### Social Media Use

Two dog-based factors significantly affected self-reported use of social media during break times for employees who took their dog to work, including dog breed *F*_(2, 242)_ = 3.73, *p* < 0.03, η_p_^2^ = 0.03, and length of time dog owned *F*_(6, 242)_ = 2.67, *p* < 0.02, η_p_^2^ = 0.06. Owners of mixed breed dogs (*n* = 35) spent significantly less time using social media during break time than owners of single (pure) breed dogs (*n* = 161) (*p* < 0.03) (see Table 4). *Post hoc* comparisons with the length of time dog owned were not significant, but there appeared to be a possible trend toward lower use of social media the longer the dog had been owned.

#### Work Related Quality of Life

Dog weight significantly affected facets of work-related QoL, including work conditions *F*_(3, 242)_ = 3.89, *p* < 0.01, η_p_^2^ = 0.05, and overall work QoL *F*_(3, 242)_ = 4.01, *p* < 0.001, η_p_^2^ = 0.05, with significantly higher QoL for employees who took their dog to work if the dog was smaller (5 kg: *n* = 18) compared to larger (21 kg: *n* = 88) (*ps* < 0.03). A similar effect was observed for the general wellbeing facet *F*_(3, 242)_ = 3.05, *p* < 0.04, η_p_^2^ = 0.04, but *post hoc* comparisons were not significant (see Table 5).

**Table 5:** Descriptive statistics for work related quality of life in employees who take their dog to their office as a function of dog-based demographics.

	**General wellbeing**	**Home-work interface**	**Job-career satisfaction**	**Control at work**	**Working conditions**	**Stress at work**	**Overall work QoL**
**Dog factors**	(Mean ± SEM)						
**Age**							
<6 months	3.25 ± 0.50	3.50 ± 0.60	4.00 ± 0.48	3.70 ± 0.58	4.35 ± 0.47	2.25 ± 0.73	3.50 ± 0.62
6 months−1 year	3.85 ± 0.15	4.04 ± 0.18	3.92 ± 0.14	4.20 ± 0.17	4.23 ± 0.14	3.11 ± 0.22	3.87 ± 0.18
1–2 years	3.75 ± 0.09	3.98 ± 0.11	3.97 ± 0.09	3.94 ± 0.10	4.20 ± 0.08	2.66 ± 0.13	4.08 ± 0.11
3–5 years	3.75 ± 0.08	3.99 ± 0.10	3.92 ± 0.08	3.85 ± 0.09	4.10 ± 0.08	2.72 ± 0.12	3.92 ± 0.10
6–8 years	3.80 ± 0.12	3.82 ± 0.15	3.89 ± 0.12	3.72 ± 0.14	4.01 ± 0.11	2.78 ± 0.18	3.94 ± 0.15
9–11 years	3.73 ± 0.12	3.73 ± 0.15	3.83 ± 0.12	3.93 ± 0.14	4.18 ± 0.11	2.77 ± 0.18	3.88 ± 0.15
12 years plus	3.85 ± 0.20	3.65 ± 0.24	3.80 ± 0.19	3.82 ± 0.23	4.02 ± 0.18	2.77 ± 0.29	3.92 ± 0.25
**Breed type**							
Single breed	3.72 ± 0.06	3.88 ± 0.07	3.91 ± 0.05	3.92 ± 0.06	4.12 ± 0.05	2.70 ± 0.08	3.91 ± 0.07
Single cross	3.76 ± 0.10	4.12 ± 0.12	3.99 ± 0.10	3.91 ± 0.12	4.16 ± 0.10	2.86 ± 0.15	4.06 ± 0.1
Mixed	3.95 ± 0.12	3.76 ± 0.14	3.80 ± 0.11	3.77 ± 0.14	4.17 ± 0.11	2.87 ± 0.17	3.97 ± 0.15
**Sex and neuter status**							
Female entire	3.88 ± 0.12	4.05 ± 0.15	4.09 ± 0.12	4.13 ± 0.14	4.29 ± 0.12	2.70 ± 0.18	3.94 ± 0.16
Female neutered	3.71 ± 0.08	3.83 ± 0.10	3.88 ± 0.08	3.84 ± 0.09	4.13 ± 0.08	2.71 ± 0.12	3.94 ± 0.10
Male entire	3.75 ± 0.10	3.95 ± 0.12	3.87 ± 0.10	3.99 ± 0.11	4.07 ± 0.09	2.74 ± 0.15	3.86 ± 0.12
Male neutered	3.78 ± 0.08	3.91 ± 0.09	3.90 ± 0.07	3.80 ± 0.09	4.12 ± 0.07	2.82 ± 0.11	4.02 ± 0.10
**Length owned**							
<6 months	3.91 ± 0.16	3.82 ± 0.20	3.97 ± 0.16	3.86 ± 0.19	4.15 ± 0.16	2.89 ± 0.24	4.11 ± 0.21
6 months−1 year	3.79 ± 0.12	3.93 ± 0.15	3.87 ± 0.12	3.97 ± 0.15	4.16 ± 0.12	2.97 ± 0.19	3.81 ± 0.16
1–2 years	3.69 ± 0.09	3.99 ± 0.11	3.92 ± 0.09	3.96 ± 0.11	4.19 ± 0.09	2.65 ± 0.14	4.04 ± 0.12
3–5 years	3.70 ± 0.08	3.98 ± 0.10	3.91 ± 0.08	3.79 ± 0.10	4.09 ± 0.08	2.70 ± 0.12	3.89 ± 0.10
6–8 years	3.85 ± 0.12	3.66 ± 0.15	3.86 ± 0.12	3.76 ± 0.14	3.97 ± 0.12	2.71 ± 0.18	3.79 ± 0.15
9–11 years	3.68 ± 0.14	3.77 ± 0.17	3.81 ± 0.14	4.01 ± 0.17	4.23 ± 0.14	2.69 ± 0.21	4.04 ± 0.18
12 years plus	4.35 ± 0.25	4.25 ± 0.30	4.39 ± 0.24	4.51 ± 0.29	4.41 ± 0.23	3.19 ± 0.37	4.50 ± 0.31
**Training**							
General obedience (GB)	3.83 ± 0.08	3.87 ± 0.09	3.97 ± 0.07	3.93 ± 0.09	4.17 ± 0.07	2.77 ± 0.11	4.04 ± 0.09
Kennel club (KC)	3.47 ± 0.23	3.84 ± 0.29	3.88 ± 0.22	3.77 ± 0.27	3.97 ± 0.22	2.61 ± 0.34	4.00 ± 0.29
Assistance dog (AD)	3.22 ± 0.28	3.82 ± 0.35	3.68 ± 0.27	3.33 ± 0.33	3.72 ± 0.27	2.92 ± 0.42	3.17 ± 0.35
Agility (Ag)	3.70 ± 0.70	3.70 ± 0.86	3.70 ± 0.67	4.30 ± 0.81	4.70 ± 0.66	2.00 ± 1.03	5.00 ± 0.86
Working dog (WD)	3.98 ± 0.25	4.29 ± 0.30	4.06 ± 0.24	4.09 ± 0.29	4.38 ± 0.23	2.69 ± 0.36	3.88 ± 0.31
Other	3.72 ± 0.28	3.57 ± 0.35	3.62 ± 0.27	3.55 ± 0.33	3.77 ± 0.27	2.67 ± 0.42	4.17 ± 0.35
None	3.71 ± 0.09	3.97 ± 0.11	3.83 ± 0.08	3.90 ± 0.10	4.14 ± 0.08	2.66 ± 0.13	3.89 ± 0.11
GB + KC + Ag	4.01 ± 0.26	4.33 ± 0.32	4.19 ± 0.25	3.86 ± 0.31	4.39 ± 0.25	3.21 ± 0.39	4.57 ± 0.33
GB + Ag	3.84 ± 0.16	3.81 ± 0.19	3.97 ± 0.15	3.99 ± 0.10	4.25 ± 0.15	2.98 ± 0.23	4.15 ± 0.19
GB + Ag + WD	3.43 ± 0.26	3.27 ± 0.32	3.44 ± 0.25	3.26 ± 0.31	3.77 ± 0.25	1.93 ± 0.39	3.43 ± 0.33
Gb + WD	4.13 ± 0.25	3.91 ± 0.30	3.89 ± 0.24	3.76 ± 0.29	3.88 ± 0.23	3.25 ± 0.36	3.75 ± 0.31
GB + KC	3.75 ± 0.20	4.12 ± 0.25	3.98 ± 0.19	4.12 ± 0.23	4.17 ± 0.19	3.00 ± 0.30	4.00 ± 0.25
AD + Other	3.56 ± 0.25	4.05 ± 0.30	4.10 ± 0.24	4.25 ± 0.29	4.09 ± 0.23	2.75 ± 0.36	3.38 ± 0.31
KC + Ag	3.80 ± 0.70	4.00 ± 0.86	4.50 ± 0.67	4.00 ± 0.81	5.00 ± 0.66	1.00 ± 1.03	4.00 ± 0.86
**Weight**							
<5kg	4.05 ± 0.16	4.19 ± 0.20	4.03 ± 0.16	4.11 ± 0.19	4.44 ± 0.15	2.67 ± 0.24	4.39 ± 0.20
6–10 kg	3.72 ± 0.10	4.00 ± 0.12	3.98 ± 0.10	4.04 ± 0.12	4.23 ± 0.09	2.72 ± 0.15	4.06 ± 0.12
11–20 kg	3.87 ± 0.07	3.77 ± 0.09	3.94 ± 0.07	3.91 ± 0.09	4.19 ± 0.07	2.76 ± 0.11	4.02 ± 0.09
21 kg plus	3.62 ± 0.07	3.95 ± 0.09	3.81 ± 0.07	3.76 ± 0.09	3.97 ± 0.07	2.78 ± 0.11	3.73 ± 0.09

## Discussion

With the aim of investigating the impact of dogs in the workplace to assess work-related and dog-related outcomes for employees who “never,” “sometimes,” and “often,” take their dog to work, we successfully conducted a large online survey. The majority of respondents were female, therefore the implications and conclusions should be considered with this in mind. We found that taking dogs to work was associated with better work related outcomes, including work engagement and commitment among dog owners. Dog weight (i.e., size), breed-type, and training may be important in considering what makes an ideal office dog. These are discussed in more detail below.

### Dogs in the Office: Work-Related and Dog-Related Outcomes

In general, employees who took their dog to work reported better work-related outcomes than those who “never” took their dog to work, on all work-related outcomes, with the exception of use of social media in break times; employees who “never” took their dog to the office used social media less than those who “often” took their dog. Those who “often” took their dog appeared to experience greater benefits from bringing their dog to work compared to those who “sometimes” take their dog to work, most notably in terms of work dedication (high levels of enthusiasm and pride in working), absorption (high levels of concentration in working), friendship acuity (better work-based friendships), vigor (energy, persistence, and effort in working), home-work interface (satisfaction in accommodating family and work commitments), control at work (perceived control over decisions), working conditions (high satisfaction with the physical working environment), and general wellbeing,. Nonetheless, those that “sometimes” took their dog to work reported greater benefits than those who “never” took their dog to work, most notably in terms of work vigor and total work engagement, and satisfaction with home-work interface and working conditions. Those employees who “often” took their dog to work also realized the greatest benefits in terms of dog-related outcomes, reporting lower anxious pet attachment.

Previous literature has qualitatively identified that a concern over taking dogs in to the office is that they will cause a distraction to the working environment (38, 41). However, the quantitative data reported here do not seem to support this concern, with employees who take their dog to work reporting higher than average absorption to working, increased vigor and general work engagement. Furthermore, it appears that individuals who take their dog to work more frequently experience greater benefits than those who only “sometimes” take their dog to the office with them, particularly in terms of their reported dedication and absorption to work. This is congruent with research in the child development literature, which suggests that the presence of a friendly dog may increase motivation and attention to set tasks (8, 15, 61–63). Indeed, the biophilia hypothesis states that humans have evolved to increase attentional processes in the presence of animals (64), and while this has been thought to underlie, at least in part, some of the benefits associated with the human-animal bond and AAI (20, 65), its relevance to work-related attention in an environment that includes dogs has not been considered before.

Employees who “often” take their dog to work also reported significantly lower intention to leave their place of current employment than those who “sometimes” and “never” take their dog to work, suggesting that taking dogs regularly to the office increases long-term job commitment as well as short-term engagement with the job. The costs of staff turnover represents a considerable strain for businesses, with estimates suggesting just one departing employee can cost $48,000–121,000, by the time hiring costs and loss of productivity have been taken into account (66, 67). Therefore, encouraging employees to regularly take their dog to the office may not only increase productivity on a day-to-day basis, but also help to ensure the longevity of the workforce. This may be particularly important to consider in certain workplaces such as the UK National Health Service (NHS) with its rapidly depleting workforce (68), provided any health risk can be properly managed.

Employee engagement and commitment may be a direct effect associated with having their pet dog with them at work, but it may also, in part, be the product of having a higher perceived work quality of life. Previous studies have suggested that the presence of a friendly pet dog can reduce both perceived stress and physiological reactions associated with stress during a complex mental task, similar to that which may be experienced in an office environment (28, 69–71). Since work-based stress impacts on employee performance (44, 72), is associated with motivations to leave current place of employment (73, 74), and has implications for general health (75–77), reducing stress, and increasing general work quality of life may enable employees to reach and sustain optimal performance. However, given that scores on the “stress at work” sub-scale were not significantly different between the groups it appears that allowing dogs in the office may bring more indirect stress reducing benefits, in terms of improving perceptions of home-work interface, control at work and working conditions. These latter two points support previous research which suggests that dogs in the office improve the general atmosphere and working environment (41, 46); nonetheless, concerns that dogs reduce the quality of working conditions due to health and safety concerns or by diminishing perceptions of professionalism (39, 41) persist despite a lack of evidence to support these supposition.

We found that employees who “often” take their dog to work reported lower anxious attachment to their dog than those who “never” and “sometimes” took their dog to work. Since owners report stress at the thought of leaving their dog unattended at home whilst they visit the office (43), it may be that allowing dogs in the office reduces this anxiety, strengthening the owner-dog bond, and contributing to general feelings of increased wellbeing and quality of life.

Another psychological mechanism which may partly underlie the beneficial work-based outcomes observed here is that employees may experience greater social support when they take their dog to the office. Not only may the dog itself act as a source of companionship and ontological security (78, 79), but the presence of a dog has also been shown to increase social interactions between individuals (21, 80). Here, we observed that employees who “often” and “sometimes” took their dog to work scored high on friendship acuity within the office, whereas individuals who “never” took their dog to work scored average, being significantly lower than employees in the “often” group. This supports previous studies which suggest that a perceived benefit of allowing dogs in the office is increased social interaction (39, 41, 46, 81). Since social support is related to stress, quality of life and working performance (82–84), increasing feelings of support are likely to bring a range of work-related benefits. Interestingly, and conversely to what was expected, we identified that employees who “never” took their dog to the office used social media less during break times than employees who “often” took their dog to the office. This difference was only significant at break times, not during work hours, or when at home, suggesting that this effect may be unique to social interactions at work, rather than in general. It is not possible to determine whether the use of social media in the work-place during working hours has a positive or negative influence on employees' work and commitment. Potential positive benefits include social media providing an effective and time-efficient mechanism to connect with colleagues, which may help minimize the impact that maintaining work-based friendships has on job performance (85, 86). There is research to indicate that employees use social media to increase respect and likeability among colleagues through posting self-enhancing messages (87). From this perspective increased use of social media may help strengthen work-based friendships and improve job satisfaction. However, the regular use of social may also have potentially negative impacts on health and wellbeing (36, 88), with reduced performance reported for some work-based tasks (89), although the current study does not support this in relation to dog owners.

Previous studies have identified animal health and welfare issues as a reason for not allowing dogs in the office (41, 46). In the present study, based on an owner self-report scale, we found no evidence to support this concern, with employees across the three dogs at work reporting similar dog health. However, we believe it is important that, in both research and practice, the implications to dog health continues to be assessed in the future. Although the simple single Likert-scale rated item used in this study is useful for providing a basic assessment, more independent and rigorous tests should be used, as assessed by clinicians using a range of techniques including examination of historical reports, behavioral observation, and physiological measures.

### Dog Demographics: The Impact on Work-Related Outcomes

With the secondary aim of identifying whether certain dogs make better working companions than others, we assessed if certain dog based demographics were related to better work-related outcomes than others.

When employers are developing dogs-in-the-workplace policies an important factor to consider may be dog size. Based on these self-report findings, it appears that larger dogs may reduce total work engagement and more specifically absorption with work, compared to smaller dogs. Similarly, allowing smaller dogs in the office may bring greater improvements to work-related quality of life. Given this effect was noticed with working conditions, which includes questions such as “I work in a safe environment” and “the working conditions are satisfactory,” it may be that bigger dogs in the office create a perception of greater hazard and reduced office space which impinges on working conditions. Employers could consider revising office layouts where possible to reduce the potentially negative effects of having larger dogs in the office. However, based on the results presented here, we cannot rule out that these effects are purely explained by dog size. Results of a meta-analysis suggest that employee personality plays a substantial role in determining work engagement, in particular; positive affectivity, proactivity, conscientiousness, and extraversion (90). Since we did not assess, or control for, the influence of personality it may be that individuals with these traits are over represented in the small dog owner group.

We also identified that dog breed may be important to consider, with employees who owned mixed breed dogs in the office using less social media during break times. However, since the implications of use of social media in this study are not clear, it is uncertain at this point whether reduced use of social media brings positive effects, in terms of increasing social interaction and reducing depression, or negative effects, such as increasing the potentially negative impact of managing friendships on work productivity. Again it is not possible to decipher whether it is the unique characteristics of the dogs, or the owners that choose to have a mixed breed dog, which reduces social media use.

A final factor to consider is dog training. Individuals whose dog had received general obedience training combined with agility training and working dog training showed higher turnover intention than those whose dogs had received general obedience training, agility training and Kennel Club training. However, the number of employees in these two groups is quite small and this may reflect other specific attributes of this group rather than purely the impact of dog training. Additionally, comparison of employees whose dogs had received just Kennel Club and working dog training were not statistically significant, suggesting there is nothing specific about these two types of training which impacts on employee turnover intention.

Although we are not able to conclusively determine the causal nature and implications of these results, we have identified that dog size, breed and training represent important factors to consider in the future and pursuing research in this area may be important if we are to develop potentially economically valuable policies.

### Characteristics of the Sample and Their Associations With Taking Your Dog to Work

Three office-based characteristics were associated with the extent to which employees took their dog to work “never,” “sometimes,” or “often”; type of organization, length of time in current employment and number of people in the office. These results suggest that working for a not-for-profit organization was associated with more employees taking their dog to work more frequently and working in the education sector was associated with taking your dog to work less frequently. This perhaps reflects that in the education sector it is considered a greater risk to health and safety to have a dog in a classroom-type environment, where there may be large numbers of young children, or more vulnerable individuals. However, there is a literature to indicate the potential promise of using dogs in educational settings, to improve behavior, literacy, memory and categorization tasks (9, 15, 91, 92). Therefore, it may be valuable to consider ways to address how to implement dogs in the classroom, whilst safeguarding both child and animal. Given that the categories used to define “type of organization” were broad, future research should consider using more refined categories.

The second factor to consider here is length of time in current employment, with employees who had been employed for longer bringing their dog to work more frequently. However, it is not clear whether this reflects an organization's willingness to allow employees who have been there longer to bring their dog to work with them, or whether being allowed to bring your dog to work influences commitment to the organization.

The third factor associated with regularly bringing your dog to work was small offices (fewer employees). This may be because it is easier to implement dogs in the workplace policies when office numbers are small, due to space restrictions and monitoring for employee allergies/fears and phobias. One dog-based factor was associated with the frequency in which dogs were taken to work—dog training. More employees who “often” took their dog to work had a dog who had received assistance dog (*n* = 5) or working dog training (*n* = 7) (e.g., shepherding or gun dog training). It is plausible that dogs who have received this arguably more intensive training were more likely to pass any organizational assessments to be allowed into the office, or that their owner or employer believed these dogs possessed the characteristics that were needed to make a successful office dog. However, since we did not assess these factors it is not possible to confirm these speculations at present.

### Study Limitations and Future Research

The majority of our respondents were female. Whilst females were represented similarly across the three dog at work groups, and therefore should not bias the results, it should be noted that our conclusions are drawn from a primarily female sample. Previous research exploring the impact of dogs in the workplace are also based on a predominantly female sample (28, 39, 42), or gender bias is not recorded (40). Studies in other fields of research also report a strong female gender bias in response rates (93, 94), suggesting this is not unique to the current topic. Future research should employ strategies to actively engage more male responders, including the targeting of male-dominated offices or sports and social clubs.

Although this study represents the largest (known) investigation to date into the impact of dogs in the office on work-related and dog-related outcomes, achieving our relatively large sample size was facilitated through the use of self-report data using, where possible, validated scales of assessment. This leaves open the possibility of a self-report bias in data collection. To help mitigate the potential pitfall with this approach, respondents were assured all responses would remain confidential and no personal identifying information was collected, however, it is still plausible that respondents felt uncomfortable answering the questions, perhaps particularly relating to turnover intention, with full honesty. Nonetheless, even if this did bias responses to a degree, we still observed significant differences between the groups. Future research should focus on collecting additional, arguably more objective, measures of work-related outcomes in offices where employees do and do not take their dog to work. Such measures should include staff retention/turnover and sick days. Sick leave represents a substantial cost to all businesses; the greatest contributing factor to the cost of mental health illnesses such as depression, is loss of work productivity from both absenteeism and presenteeism (working whilst dealing with depression), collectively estimated to cost around $250 billion/year across eight countries, (4). Other estimates, taking into account broader aspects of loss (not unique to business productivity), show that in 2010 the worldwide cost of mental health issues were estimated at $2.5–8.5 trillion (6) and this is likely to have increased significantly since then and can be expected to continue to do so (95). Since dog companionship may offer some protection against depression (96–99), there is a strong argument for investigating whether allowing dogs in the workplace reduces both absenteeism and presenteeism due to depression. Work absence due to pet care issues and its impact should also be considered.

## Conclusion

The results add to the growing body of work that indicates the potential benefits of encouraging owners to take their dogs to work, highlighting their value in employee engagement and commitment to work, work-related quality of life and work-based friendships, in our predominantly female sample. However, we wish to stress that it is likely that the potential benefits of allowing dogs in the office will only be maximized through the implementation of well-designed policies, which have been developed with the input from multi-disciplinary teams incorporating organizational psychologists and animal behaviorists. Previous research by Hall et al. (41) may prove a useful starting point in the development of such policies. We report, for the first time, some of the potential dog-based factors which may be important to consider in the development of these policies, notably dog size and dog breed-type. Nonetheless, future research is required to substantiate the relative importance of these factors.

## Data Availability

All datasets generated for this study are included in the manuscript and/or the supplementary files.

## Ethics Statement

The study was carried out in accordance with the recommendations of the British Psychological Society (BPS) Ethical Code of Conduct, with written informed consent from all subjects. All subjects gave written informed consent in accordance with the Declaration of Helsinki. The protocol was approved by the University of Lincoln's College of Science Research Ethics Committee (ID: CoSREC367).

## Author Contributions

SH was responsible for co-designing the study, data collection, analysis, interpretation and manuscript preparation. DM was responsible for co-designing the study, statistical support, data interpretation and manuscript preparation.

### Conflict of Interest Statement

The authors declare that the research was conducted in the absence of any commercial or financial relationships that could be construed as a potential conflict of interest.
